# Does Leaving the Biopsy Needle in Povidone-Iodine Solution Reduce Infective Complications after Biopsy?

**DOI:** 10.1155/2016/6841837

**Published:** 2016-12-20

**Authors:** Erdal Benli, Abdullah Cirakoglu, Ercan Ogreden, Yeliz Cetinkol, Mustafa Kerem Calgin, Ali Ayyildiz, Ahmet Yüce

**Affiliations:** ^1^Department of Urology, Faculty of Medicine, Ordu University, Ordu, Turkey; ^2^Department of Urology, Faculty of Medicine, Giresun University, Giresun, Turkey; ^3^Department of Microbiology, Faculty of Medicine, Ordu University, Ordu, Turkey

## Abstract

*Aim*. The aim of this study is to evaluate whether leaving the biopsy needle used during prostate needle biopsy in 10% povidone-iodine (betadine) solution affects the infectious complications forming after biopsy.* Material and Method*. This study retrospectively evaluated the data of 176 patients with prostate biopsy performed between December 2012 and April 2014. Patients in Group 1 (*n* = 89) were given ofloxacin as a prophylactic antibiotic before biopsy. Patients in Group 2 (*n* = 87) had the biopsy needle left in povidone-iodine solution for 1 minute before each use, in addition to antibiotic prophylaxis. The two groups were compared in terms of infective complications developing after biopsy. Results were analyzed using the Mann–Whitney *U* test and Fisher's exact test.* Results*. The distribution of infective complications after biopsy according to group was as follows. Group 1, not using betadine, had 15.7% fever, 13.5% hospital stay, 12.4% urinary retention, 10.1% prostatitis, and 5.6% sepsis. The distribution of the same complications in Group 2 using betadine was identified as 5.7% fever, 4.6% hospital stay, 3.4% urinary retention, 2.3% prostatitis, and 0% sepsis. The use of betadine was found to significantly reduce the infectious complications after biopsy compared to the control group (*p* < 0.05).* Conclusion*. At the end of this study leaving the prostate needle in povidone-iodine solution before each use during prostate biopsy was found to reduce the infective complications and hospital stay after biopsy.

## 1. Introduction

Prostate needle biopsy (PNB) accompanied by transrectal ultrasound is an essential diagnostic tool for the diagnosis of prostate cancer. Though PNB is a safe and well-tolerated procedure, after the procedure temporary complications such as hematuria, rectal bleeding, and dysuria may frequently occur, while significant complications like prostatitis and sepsis may rarely occur [1]. To prevent these complications it is recommended that the biopsy procedure be completed with antibiotic prophylaxis [2].

In spite of quinolone prophylaxis, infective complications are observed at rates of up to 36% related to the biopsy procedure [3]. It is thought that these infections are caused by the increasing incidence of quinolone-resistant bacteria [4, 5]. To reduce complications after biopsy, additional methods of intestinal preparation such as the use of antibiotics based on rectal swabs and enema are used. There is debate about the benefits of intestinal preparation. Some studies have shown that intestinal preparation reduces infective complications [6, 7], while others have not found additional benefit [8]. It is known that bacteria in the colon may be carried on the biopsy needle through tissues and the urinary tract causing infections [9]. From the first moment the needle used for biopsy enters the rectum, it is contaminated by intestinal contents and we believe that this dirty needle is responsible for complications occurring. According to our hypothesis, leaving the needle in betadine solution during the procedure will reduce the infective complications occurring after biopsy.

The aim of this study is to investigate whether leaving the prostate biopsy needle in 10% povidone-iodine solution for 1 minute before each use has an effect on infective complications after biopsy.

## 2. Material and Methods

Patients attending Ordu University Faculty of Medicine between April 2012 and March 2015 with prostate biopsy performed were retrospectively investigated. For all patients the first biopsy results were used.

Patients attending before December 2013 were given total of 3 doses of 200 mg quinolone 2 hours before biopsy and 2 and 12 hours after biopsy (Group 1). Quinolones reach effective plasma concentrations 1–3 hours after oral intake. Additionally it is known there may be some slowing of absorption of medication in older patients. As a result, instead of administering the medication 1 hour before the procedure, as is common in the literature, we administered the medication 2 hours before the procedure. Apart from this no additional intestinal preparation was administered. After observing high infection rates and serious sepsis cases, our biopsy technique was reviewed and the decision was made to disinfect the biopsy needle, considered to be the source of infection, by leaving it in betadine. From December 2013 to March 2015 in addition to the antibiotic regime of 3 doses of 200 mg ofloxacin, during the procedure the biopsy needle was left for 1 minute in a container designed to fully cover the needle containing povidone-iodine solution (PVP-I/Betadine®). During the whole procedure the same solution was used, and the needle was fully wiped with a betadine sponge before and after being placed in betadine. The same procedure was repeated after each tissue sample was obtained (Group 2). Before biopsy procedures no intestinal preparation using betadine or enema was performed. The biopsy procedure was completed as a sextant biopsy in left lateral decubitus position, using a 22-gauge needle accompanied by transrectal ultrasound.

The study had exclusion criteria of prostatitis or sexually transmitted disease history in the previous month, use of permanent ureteral probe, antibiotic use during biopsy, full rectum or anorectal surgery like hemorrhoid or anal fistula in the previous month, and history of colon or anal cancer. Patients using anticoagulants had cardiology consultation and according to the cardiologist's recommendation medications were ceased at least 7 days before. No patient had biopsy performed while taking anticoagulants. Patients with prostate specific antigen (PSA) levels above 4 ng/dL or with suspicious rectal examination findings and those patients who gave written consent for the procedure had biopsy performed.

The patients' age, waist circumference, additional diseases, previous biopsy, PSA levels, prostate volume, and amount of residual urine were recorded. Complications relating to the biopsy procedure were explained in detail to the patients and a signed consent form was provided by the patients. All patients were given the same antibiotic protocol. Before the biopsy procedure 2% lidocaine was used (2 cc bilaterally) for periprostatic nerve block. A 12-core biopsy was performed accompanied by transrectal ultrasound.

After the biopsy procedure, to monitor possible complications in patients that may not reach our clinic in time if complications occurred, the patient was admitted to hospital for 1 night. All patients were reminded to return to our clinic if they encountered any problems such as fever, shivering, or bleeding after the procedure and were discharged without prescription. All patients were called for a check-up 7 days after the procedure. Clinically significant complications were accepted as requiring treatment not normally administered or requiring hospital stay due to symptoms. Infection was defined according to Harrison's principle of internal medicine as fever above 37.7°C within 1 week after biopsy or observation of shivering [10]. Patients applying to the hospital for complications after biopsy had temperature reading and urine and blood cultures obtained by a nurse. Patients applying with the complaint of not being able to urinate and with swollen bladder on physical examination were accepted as having retention. For diagnosis of prostatitis, painful urination, difficulty emptying the bladder, pelvic region pain, fever, and shivering were used. For diagnosis of sepsis, general situation disorder, high fever, increased heart rate, increased respiratory rate, and infection documented in blood were used. All patients were met with pathology results within 15 days at most.

## 3. Statistical Analysis

Results were analyzed using the Mann–Whitney *U* test and Fisher's exact test. Significance between parameters used *p* < 0.05 as criterion.

## 4. Results

The mean age and age interval of patients included in the study in Group 1 and Group 2 were 65.4 ± 8.7 (41–88) and 65.3 ± 8.6 (48–86) years, respectively. There were 89 patients in Group 1 and 87 patients in Group 2. The antibiotic prophylaxis regime, number of biopsies, and biopsy technique were similar in the groups. The characteristics of the groups are shown in Table 1. The distribution of infective complications after biopsy in the groups was as follows, as shown in Table 2. In Group 1, 14 patients had fever (15.7%) and 9 patients had prostatitis (10.1%), while in Group 2 these complications were found in 5 (5.7%) and 2 (2.3%) patients, respectively (*p* = 0.033 and *p* = 0.032), while sepsis was observed in 5 patients (5.6%) in Group 1; no patient in Group 2 developed sepsis (*p* = 0.025). Urinary retention linked to prostatitis occurred in 11 patients (12.4%) in Group 1 and in 3 patients (3.4%) in Group 2. The rate of urinary retention requiring catheterization in Group 1 was observed to be significantly high compared to Group 2 (*p* = 0.029). The patient numbers requiring hospitalization were 12 (13.5%) and 4 (4.6%) patients in Groups 1 and 2, respectively (*p* = 0.04). After biopsy the rate for all complications in the groups were 26 patients in Group 1 (29.2%) and 7 patients in Group 2 (8%). In terms of complication rates, the difference between the two groups was statistically significant (*p* < 0.001).

The distribution of bacteria obtained from urine and blood cultures belonging to patients developing infective complications after PNB is shown in Table 3. In the 26 patients in Group 1 with infective complications, 15 (57%) had* E. coli* identified in urine culture, while 5 (19%) had* E. coli* identified in blood cultures. In the 7 patients in Group 2 with infective complications, 5 (71%) had* E. coli* identified in urine cultures, while there was no proliferation observed in blood cultures.

## 5. Discussion

This study found that the use of a biopsy needle left in povidone-iodine solution significantly reduced infective complications after biopsy such as fever, prostatitis, and sepsis, as well as hospital stay linked to these complications, compared to a control group.

The common use of PSA for prostate cancer screening has increased the number of biopsies performed. It is estimated that the number of biopsies performed per year in the United States of America is more than 1 million [11]. Though the biopsy procedure is accepted as a reliable, generally outpatient, procedure, a variety of infective complications may occur after PNB from asymptomatic pyuria to life-threatening sepsis [12, 13]. To prevent these complications a variety of antibiotic groups are commonly used. Among these antibiotics, due to characteristics such as broad antibacterial spectrum, high bioavailability, and reaching high concentrations in tissues such as the prostate, quinolones comprise a frequently chosen antibiotic group [14]. A study by Puig et al. showed that biopsy performed with antibiotic prophylaxis reduced infective complications occurring after PNB compared to a group not given antibiotics (3.7% compared to 10.3%) [15].

In spite of biopsy procedures being performed with antibiotic prophylaxis, it is known that some patients (about 2.5%) will apply to hospital with serious complications [16]. The increasing incidence of quinolone-resistant bacteria is held responsible for the development of these infections. A study by Livermore et al. identified that the incidence of quinolone-resistant* E. coli* increased 7 times between 1990 and 1999 (0.8% compared to 3.7%) [17]. Zaytoun et al. found quinolone-resistant* E. coli* in urine and blood cultures of 55% of patients developing infective complications after PNB [18]. Similar to our results, in the study by Pinkhasov et al.* E. coli* was most frequently identified (92%) in infective complications occurring after PNB and quinolone resistance was reported as 75% [16]. Another study similarly found* E. coli* was the most common proliferative agent in urine cultures (92%) and reported quinolone resistance as 83% [19].

One of the methods investigated to reduce these infective complications is antibiotic use based on rectal swabs. When the most appropriate antibiotic for bacteria identified on rectal swabs is administered, it is expected that infections will not occur. There is controversy related to the use of antibiotics based on rectal swabs. Some studies have reported a reduction in infective complications compared to control group [20, 21], while others have shown no benefit [22]. Due to debates about efficacy, loss of time, and increased costs, we do not use rectal swabs in our clinic.

Another method investigated to reduce infective complications after biopsy is the use of enema for intestinal preparation. Some studies have reported a reduction in infection rates compared to control group [6], while other studies have shown no benefit [8, 23]. The requirements for assisting personnel for the procedure, in addition to cost and provision of additional discomfort to already concerned patients, are disadvantages of enema. In European and American guidelines, the routine use of enema is not recommended [24]. In our clinic enema is not used.

Disinfecting the colon with povidone-iodine before biopsy is another additional method studied to reduce infective complications. Povidone-iodine is an easily accessed, cheap antiseptic solution commonly used to clean the surgical field for daily surgical procedures. As it is safe for mucosa, it is commonly used during gynecological and colorectal surgery [25]. A study by Park et al. divided 408 patients undergoing biopsy into two groups of patients either administered povidone-iodine in suppository form for intestinal preparation or not. In the group with intestinal preparation the infective complication rate was 0.3%, while in the control group this rate was 6.6%. The results of the study found that intestinal preparation with povidone-iodine reduced infective complications and the number of colonies in the colon [26]. However, other studies did not find any difference between control group and groups with intestinal disinfection using povidone-iodine. In the study by Abughosh et al. 865 biopsy patients were divided into two groups with one given intestinal preparation using povidone-iodine and the other control group without intestinal preparation. The results of the study reported no difference between the groups in terms of infective complications [27].

Similar to our study, the study by Koc et al. washed the biopsy needle used for the biopsy procedure in povidone-iodine solution before each use. The results of the study reported that no difference was found between the povidone-iodine group and control group in terms of infective complications [28]. The results obtained in that study do not comply with our study results. We believe the reason for this is that the biopsy needle was not in contact with the solution for sufficient time. For needle biopsy, as in our study, we believe that when the needle is left in betadine solution for sufficient time (e.g., 1 minute in our study) it may reduce the infective complications like fever, sepsis, and prostatitis that occur after biopsy.

There are some limitations to our study. The first is that the study was retrospective, nonrandomized, and single centered. Another limitation of the study is that it does not include a recent history of antibiotic use by patients. As a result, we believe more comprehensive studies are required to evaluate the presented technique. However, to the best of our knowledge, this is the first study to present this new technique to the literature, and as the results are interesting we believe it is important. Some of the most important advantages of the presented technique are that it is simple, easy to access, and does not cause additional burden to the patient.

## 6. Conclusions

This study found that leaving the biopsy needle in povidone-iodine solution for disinfection reduced infective complications after biopsy and hospital stay linked to complications compared to a control group. This study has the distinction of being the first in the literature, to our knowledge, to examine the results of disinfecting the biopsy needle by leaving it in betadine solution for 1 minute. This effect of povidone-iodine may be due to reducing the number of bacteria on the biopsy needle later carried into prostate tissue, veins, and urinary tract. The needle is sterile at the beginning of the procedure, but after the first use sterility is lost, so to avoid infective complications the amount of bacteria carried into tissue on the biopsy needle should be reduced. We believe betadine may serve this purpose. However, our results should be supported by more comprehensive and multicenter studies.

## Figures and Tables

**Table 1:** Characteristics of patients in the groups.

Characteristic	Group 1 (*n* = 89)	Group 2 (*n* = 87)	*p* value
Age (y), mean ± SD (range)	65.4 ± 8.7 (41–88)	65.3 ± 8.6 (48–86)	0.812
Diabetes mellitus *n* (%)	18 (20.2)	25 (28.7)	0.221
Waist circumference *n* (%)	39 (43.8)	45 (5.7)	0.294
Hypertension *n* (%)	25 (28.1)	30 (34.5)	0.360
Metabolic syndrome *n* (%)	32 (36)	35 (40.2)	0.559
Heart disease *n* (%)	18 (20.2)	15 (17.2)	0.612
Smoker (%)	35 (39.3)	33 (37.9)	0.849
LUTS *n* (%)	31 (34.8)	35 (40.2)	0.460
Previous history of biopsy *n* (%)	18 (20.2)	17 (19.5)	0.909
PSA level (ng/mL), median (min–max)	7.20 (2.28–100)	6.70 (0–150)	0.293
Prostate volume median (min–max)	50 (13–153)	45 (8–131)	0.224
PVR median (min–max)	15 (0–300)	15 (0–433)	0.494

**Table 2:** Distribution of complications according to groups.

Characteristic	Group 1 (*n* = 89)	Group 2 (*n* = 87)	*p* value
Fever *n* (%)	14 (15.7)	5 (5.7)	0.033
Temperature (°C) (mean ± SD) (range)	38.7 ± 0.9 (37.7–40.2)	39.3 ± 0.6 (38.7–40)	0.22
Sepsis *n* (%)	5 (5.6)	0 (0)	0.025
Prostatitis *n* (%)	9 (10.1)	2 (2.3)	0.032
Urinary retention *n* (%)	11 (12.4)	3 (3.4)	0.029
Hospital stay *n* (%)	12 (13.5)	4 (4.6)	0.040
General complications *n* (%)	26 (29.2)	7 (8)	<0.001

**Table 3:** Distribution of bacteria in urine and blood culture according to groups.

Characteristic	Group 1 (*n* = 26)	Group 2 (*n* = 7)
Urine culture		
*Escherichia coli* (%)	15 (%57)	5 (%71)
Quinolone resistance (%)	10 (%66.6)	4 (%80)
ESBL (%)	6 (%40)	3 (%60)
Blood culture		
*Escherichia coli* (%)	5 (%19)	0
Quinolone resistance (%)	5 (%100)	0
ESBL (%)	4 (%80)	0

ESBL, extended spectrum beta-lactamase.
